# Bioactive Phytochemicals and Functional Food Ingredients in Fruits and Vegetables

**DOI:** 10.3390/ijms21093278

**Published:** 2020-05-06

**Authors:** Francesca Giampieri, Maurizio Battino

**Affiliations:** 1 Nutrition and Food Science Group, Department of Analytical and Food Chemistry, CITACA, CACTI, University of Vigo—Vigo Campus, 32004 Ourense, Spain; f.giampieri@univpm.it; 2 Dipartimento di Scienze Cliniche Specialistiche e Odontostomatologiche—Università Politecnica delle Marche, Via Ranieri 65, 60130 Ancona, Italy; 3 College of Food Science and Technology, Northwest University, Xi’an 710069, China; 4 International Research Center for Food Nutrition and Safety, Jiangsu University, Zhenjiang 212013, China

Today, it is widely accepted that a plant-based diet produces wellbeing and prevents the onset of several human diseases [1,2,3,4,5]. In recent years, fruits and vegetables, thanks to their high contents and variety of bioactive compounds, have attracted the attention of the scientific community worldwide, as demonstrated by pivotal papers that have obtained a very high number of citations and that currently represent milestones in this field [6,7,8,9,10,11,12,13,14,15]. *International Journal of Molecular Sciences* has always emphasized this interesting topic: the Special Issue “Bioactive Phytochemicals and Functional Food Ingredients in Fruits and Vegetables” was first written in 2015, obtaining significant success with 31 published papers, and the same successful results were gained in subsequent editions in 2016 and 2017. For these reasons, we decided to update these topics in 2019 too, where ten outstanding papers, from experts in this field, provided a broad range of contributions, describing different aspects of polyphenol health benefits and highlighting potential mechanisms involved in their positive effects.

The protective effects of dietary bioactive compounds and polyphenols against oxidative stress and inflammation, two processes that are implicated in the pathogenesis of several common diseases, have been widely evaluated in recent years [10,16,17,18]. In this Special Issue, six research papers were assessed and confirmed these beneficial properties: for example, Kim et al. showed that pachypodol, a 4′,5-dihydroxy-3,3′,7-trimethoxyflavone isolated from *Pogostemon cablin* Bentham (patchouli), exerts antioxidant activity and cytoprotective effects in HepG2 cells stressed with tert-butylhydroperoxide, decreasing intracellular reactive oxygen species through the phosphorylation of ERK and the consequent activation of the Nrf2/ARE pathway [19]. At the same time, the 3,5-dihydroxy-4′,7-dimethoxyflavone, isolated from *Tamarix aphylla* L., has been shown to ameliorate histopathological changes, suppress oxidative stress, enhance the antioxidant system, and decrease apoptosis and angiogenesis in the liver of carbon tetrachloride-treated mice [20]. Moreover, the paper of Dicarlo et al. found that quercetin is capable of inhibiting inflammation in ulcerative colitis and in lipopolysaccharide-treated wild-type organoids, decreasing the gene expression of the main inflammatory markers, such as TNF-α and LCN-2 [21], while Ginger berry suppresses inflammation and increases antioxidant enzyme activities in lipopolysaccharidetreated macrophages and in ethanol-treated mice, thus preventing alcohol-induced liver damage, as demonstrated by Lee et al. [22]. Similarly, Hwang et al. showed that Zerumbone, a natural compound of the *Zingiber zerumbet* (L.) Smith plant, decreases colonic inflammation, reducing the expression of TNF-α, IL-17A and inducible nitric oxide synthase, through the inhibition of NF-κB signaling pathway in a murine model of enterotoxigenic *Bacteroides fragilis* infection [23], while Curti et al. revealed that in C57BL/6 wild-type mice propolis, after being rapidly absorbed and metabolized, increases antioxidant defense system, especially SOD-1 expression [24].

In addition, two research papers have proved the hypolipidemic effects of natural compounds and plant food: on one hand, shikimic acid, a natural compound isolated from *Illicium verum,* attenuates lipid accumulation and de novo lipogenesis by reducing the gene expression of fatty acid synthase, sterol regulatory element-binding protein 1c, and LXR-α through the phosphorylation of the AMP-activated protein kinase (AMPK)/ acetyl CoA carboxylase pathway in HepG2, Huh7, and 3T3-L1 cells [25]; on the other hand, the mushroom *Poria cocos* Wolf improves hepatic steatosis by regulating lipid metabolism, inhibiting endoplasmic reticulum stress, and inducing the autophagic machinery through AMPK activation in HepG2 cells and in obese mice [26].

Several natural compounds are promising candidates to counteract microbial infections and overcome the crucial global concern of antibiotic resistance. Chang et al. stated that Tellimagrandin II, a polyphenol extracted from the shells of *Trapa bispinosa,* has strong inhibitory activity against methicillin-resistant *Staphylococcus aureus* (MRSA), by reducing the expression of *mecA* and the consequent negative regulation of MRSA penicillin-binding protein 2a [27].

Finally, functional foods can act as an anticancer agents against different types of tumors [28,29]. This is described, for example, in the work of Aryappalli et al., who found that Manuka honey inhibits p-STAT3, decreasing the levels of gp130 and p-JAK2 binding directly to IL-6 receptor α in breast (MDA-MB-231) and lung (A549) cancer cell lines [30].

In summary, all papers published in this Special Issue show the health benefits of plant bioactive compounds and functional foods, ranging from antioxidant, anti-inflammatory, and antimicrobial capacities to hypolipidemic and anticancer effects, to reveal the molecular mechanisms involved. We want to thank all the authors for their interesting contributions, which offer different insights into the multitargeted effects of natural compounds, allowing the readers to update their knowledge on the described mechanisms in this extremely complex field.
